# Drug Prescribing Patterns in Geriatric Patients With Type 2 Diabetes Mellitus at a Tertiary Care Teaching Hospital: A Cross-Sectional Study

**DOI:** 10.7759/cureus.104264

**Published:** 2026-02-25

**Authors:** Deeksha Gupta, Anurag Sharma, Sartaj Hussain, K K Sawlani, Devendra Katiayar, Kauser Usman, Rajendra Nath

**Affiliations:** 1 Pharmacology and Therapeutics, King George's Medical University, Lucknow, IND; 2 Pharmacology, All India Institute of Medical Sciences, Vijaypur, IND; 3 Internal Medicine, King George's Medical University, Lucknow, IND

**Keywords:** drug prescribing patterns, geriatric diabetes, polypharmacy, rational drug use, who core prescribing indicators

## Abstract

Introduction

Type 2 diabetes mellitus (T2DM) is highly prevalent among elderly individuals and is frequently accompanied by multiple comorbidities, resulting in complex pharmacotherapy and an increased risk of polypharmacy and irrational prescribing. Evaluating real-world prescribing patterns using standardized indicators is essential to promote rational drug use and optimize therapeutic outcomes in geriatric patients with T2DM.

Methods

A hospital-based, cross-sectional observational study was conducted over one year (April 2024 to March 2025) in the outpatient department of a tertiary care teaching hospital in North India. A total of 600 geriatric patients (≥60 years) with T2DM were included. Demographic and clinical data were recorded using a predesigned case record form. Prescriptions were analyzed using World Health Organization (WHO) core prescribing indicators. Polypharmacy was defined as the concurrent use of five or more medications. Descriptive statistics were used to summarize the data.

Results

The mean age of the study population was 66.4 ± 4.8 years, with a male predominance (63%). Hypertension (68%) was the most common comorbidity, followed by chronic kidney disease (35.1%) and dyslipidemia (18.2%). The average number of drugs per prescription was 5.22 ± 1.37, indicating a high prevalence of polypharmacy. Drugs were prescribed by generic name in 57.7% of cases, and injectable medications were used in 24.2% of encounters. The mean number of antibiotics prescribed per encounter was 0.44 ± 0.81. Only 21% of the prescribed drugs were from the National List of Essential Medicines. Sulfonylureas were the most commonly prescribed antidiabetic drug class, and dual antidiabetic therapy was the most frequent regimen (42%).

Conclusion

Geriatric patients with T2DM attending a tertiary care hospital experience a high burden of polypharmacy, reflecting the presence of multiple comorbidities. Although moderate generic prescribing and judicious antibiotic use were observed, adherence to essential medicine prescribing remains suboptimal. Regular prescription audits, rational drug use policies, and periodic medication review are necessary to improve the safety, affordability, and quality of pharmacotherapy in elderly patients with T2DM.

## Introduction

Persistent hyperglycemia brought on by compromised insulin production, action, or both is a hallmark of diabetes mellitus (DM), a chronic metabolic disease. Over 90% of all instances of diabetes globally are type 2 diabetes mellitus (T2DM), which poses a serious public health concern because of its increasing incidence, long-term consequences, and high cost of care. Over the past few decades, sedentary lifestyles, urbanization, population aging, and rising obesity rates have all contributed to a sharp rise in the prevalence of diabetes worldwide [1]. In India, diabetes has emerged as a major non-communicable disease, with the country currently ranking among those with the highest number of adults living with diabetes globally [1].

The burden of T2DM is disproportionately higher among older adults. Advancing age is associated with progressive β-cell dysfunction, insulin resistance, and accumulation of cardiometabolic risk factors, making elderly individuals particularly vulnerable to diabetes and its complications [2,3]. Furthermore, geriatric patients commonly present with multiple coexisting chronic conditions such as hypertension, dyslipidemia, cardiovascular disease, and chronic kidney disease, which necessitate the use of multiple medications concurrently [4]. This complex clinical profile often results in polypharmacy, commonly defined as the use of five or more medications, which is highly prevalent among elderly patients with diabetes [5].

In older people, polypharmacy is linked to a number of negative outcomes, such as a higher chance of adverse drug reactions, drug-drug interactions, prescription non-adherence, functional decline, falls, hospitalization, and higher healthcare expenses [5,6]. Age-related changes in pharmacokinetics and pharmacodynamics - such as reduced renal clearance, altered hepatic metabolism, and changes in body composition - further increase the susceptibility of elderly patients to medication-related harm [7]. These factors highlight the importance of rational prescribing and regular review of medication regimens in elderly patients with T2DM.

Prescription pattern studies are a valuable tool for analyzing actual prescribing patterns, determining if drug use is logical, and spotting discrepancies between clinical practice and evidence-based recommendations. To encourage the prudent use of medications, the World Health Organization (WHO) has created core drug prescribing indicators. These include the average number of medications prescribed per encounter, the percentage of medications prescribed by generic names, the percentage of encounters involving injections or antibiotics, and the percentage of medications prescribed from essential medicine lists [8]. These indicators provide an objective framework for auditing prescribing practices and are widely used in drug utilization studies across diverse healthcare settings [9].

Pharmacological management of T2DM in elderly patients requires careful individualization, balancing glycemic control with safety concerns such as hypoglycemia, renal impairment, cardiovascular comorbidities, and treatment burden. Metformin is recommended as first-line therapy in most patients due to its efficacy, safety profile, and cardiovascular benefits, and is included in the WHO Model List of Essential Medicines [10,11]. In actual practice, however, a range of injectable therapies and oral antidiabetic medications are administered based on patient characteristics, physician preferences, availability, and cost. Newer classes of antidiabetic drugs, such as sodium-glucose co-transporter-2 (SGLT-2) and dipeptidyl peptidase-4 (DPP-4) inhibitors, have potential cardiovascular or renal benefits and a lower risk of hypoglycemia, but their use in older patients with comorbidities may be restricted by their cost and contraindications [12].

There is still a dearth of information on the drug prescription patterns of elderly patients with type 2 diabetes in Indian tertiary care settings, despite the high prevalence of the disease among this population and the difficulties associated with its pharmacotherapy. To identify areas that need intervention and improve the quality of care, it is essential to comprehend current prescription trends, the degree of polypharmacy, and adherence to rational prescribing indicators. Clinicians, hospital managers, and legislators can use this information to learn about current procedures and possible ways to encourage sensible, evidence-based prescribing in this susceptible group [9,13].

The primary objective of this study was to evaluate prescribing patterns in geriatric patients with T2DM using WHO core prescribing indicators. The secondary objectives were to describe the demographic and clinical characteristics of the study population, determine the prevalence of polypharmacy (defined as the prescription of five or more total medications per encounter), and assess patterns of antidiabetic drug utilization.

Therefore, the present study was undertaken to evaluate the drug prescribing patterns in elderly patients with T2DM attending a tertiary care teaching hospital, with a focus on demographic and clinical characteristics, overall antidiabetic drug utilization, polypharmacy prevalence, and assessment of rational prescribing practices using WHO core prescribing indicators.

## Materials and methods

Study design and setting

A hospital-based, cross-sectional observational study was conducted in the outpatient department (OPD) of the Department of Internal Medicine at a tertiary care teaching hospital in North India over a period of one year (April 2024 to March 2025). The study was designed to evaluate real-world prescribing practices in elderly patients with T2DM and to assess the rationality of prescriptions using standardized indicators [8,9].

Study population

The study population comprised geriatric patients aged 60 years and above diagnosed with T2DM and attending the medicine OPD during the study period. The diagnosis of T2DM was based on documented medical records and physician diagnosis in accordance with the International Diabetes Federation (IDF) 10th edition criteria applicable during the study period, including fasting plasma glucose ≥126 mg/dL, glycated hemoglobin (HbA1c) ≥6.5%, two-hour plasma glucose ≥200 mg/dL during an oral glucose tolerance test, or random plasma glucose ≥200 mg/dL in the presence of classic symptoms of hyperglycemia. Patients were recruited consecutively during routine outpatient visits to minimize selection bias and to reflect routine prescribing practices in a real-world clinical setting.

Inclusion and exclusion criteria

Patients aged ≥60 years who were diagnosed with T2DM and attending the medicine OPD during the study period were eligible for inclusion. Only those willing to participate and provide written informed consent were enrolled. Patients with type 1 DM, those requiring emergency care or hospitalization at the time of recruitment, patients with terminal illness, and those with severe cognitive impairment or psychiatric illness that could affect the ability to provide informed consent were excluded. Prescriptions with incomplete documentation were also excluded from the analysis.

These criteria were used to ensure inclusion of stable ambulatory elderly patients receiving routine outpatient care and to avoid confounding due to acute illness or emergency management [2].

Sample size and sampling technique

A total of 600 geriatric patients with T2DM were included in the study using a consecutive sampling technique over the study duration. Consecutive sampling was adopted to reduce selection bias and ensure representation of routine outpatient prescribing patterns. The sample size was considered adequate based on the patient load of the OPD and feasibility within the study period.

Ethical considerations

The study protocol was reviewed and approved by the Institutional Ethics Committee (approval no.: XXIII-PGTSC-IIA/P12). The study was conducted in accordance with the ethical principles outlined in the Declaration of Helsinki and the national ethical guidelines for biomedical and health research involving human participants [5]. Written informed consent was obtained from all participants prior to data collection. Confidentiality of patient information was strictly maintained, and no personal identifiers were recorded.

Data collection tool and procedure

Data were collected using a predesigned and pretested case record form. The form captured demographic details, including age and sex, and clinical characteristics, including the presence of comorbidities such as hypertension, chronic kidney disease, dyslipidemia, cardiovascular disease, and other relevant conditions. Prescription details were recorded for each patient encounter, including the total number of drugs prescribed, names of antidiabetic and concomitant medications, dosage forms and routes of administration, use of injectable formulations, use of antibiotics, generic versus brand name prescribing, and whether prescribed drugs were included in the National List of Essential Medicines (NLEM). Prescriptions were reviewed at the point of care without influencing the treating physician’s prescribing decisions.

Assessment of prescribing patterns and indicators

Prescribing patterns were evaluated using the WHO core prescribing indicators. These indicators included the average number of drugs per encounter, the percentage of drugs prescribed by generic name, the percentage of encounters with an injection prescribed, the mean number of antibiotics prescribed per encounter, and the percentage of drugs prescribed from the NLEM (adherence to essential medicines was assessed using the NLEM 2022. Polypharmacy was defined as the concurrent prescription of five or more medications (antidiabetic, along with concomitant medications) in a single encounter. Patterns of antidiabetic drug utilization were assessed by categorizing prescribed antidiabetic agents into major drug classes and by recording the number of antidiabetic drugs prescribed per encounter.

Statistical analysis

Data were entered into Microsoft Excel (Microsoft® Corp., Redmond, WA) and analyzed using descriptive statistical methods. Continuous variables were summarized as mean ± standard deviation (SD), while categorical variables were expressed as frequencies and percentages. Results were presented using tables and graphical representations to enhance clarity and interpretability. No inferential statistical tests were applied, as the primary objective of the study was descriptive evaluation of prescribing patterns.

## Results

Baseline demographic and clinical characteristics

A total of 600 geriatric patients with T2DM were included in the study. The mean age of the study population was 66.4 ± 4.8 years. There was a male predominance, with males comprising 378 (63%) of the study population and females 222 (37%), resulting in a male-to-female ratio of approximately 1.7:1.

The majority of patients had at least one comorbid condition in addition to diabetes. Hypertension was the most common comorbidity, present in 408 (68%) patients, followed by chronic kidney disease in 211 (35.1%) and dyslipidemia in 109 (18.2%). Other comorbidities included respiratory diseases in 48 (8%), thyroid disorders in 32 (5.3%), and cardiac conditions in 14 (2.3%). A smaller proportion of patients, 72 (12%), had other comorbidities such as musculoskeletal or gastrointestinal disorders (Table 1).

**Table 1:** Demographic and clinical characteristics of geriatric patients with type 2 diabetes mellitus Data are presented as mean ± standard deviation (SD) for continuous variables and number (percentage) (N (%)) for categorical variables.

Parameter	Value
Age ± SD	66.4 ± 4.8
Males (%)	378 (63.0%)
Females (%)	222 (37.0%)
M:F ratio	1.7:1
Comorbidities	
Hypertension	408 (68.0%)
Chronic kidney diseases	211 (35.1%)
Dyslipidemia	109 (18.2%)
Respiratory diseases	48 (8.0%)
Thyroid	32 (5.3%)
Cardiac problems	14 (2.3%)
Others	72 (12.0%)

WHO core prescribing indicators

The average number of drugs per encounter (5.22 ± 1.37) represents the total number of all prescribed medications per encounter, including antidiabetic drugs and concomitant medications. Generic prescribing constituted 57.7% of all prescribed drugs. Injectable medications were prescribed in 145 (24.2%) encounters. The mean number of antibiotics per encounter was 0.44 ± 0.81. Drugs from the NLEM constituted 21% of all prescribed drugs (Table 2).

**Table 2:** World Health Organization core prescribing indicators in the study population Data are presented as mean ± standard deviation (SD) and number (percentage) (N (%)), as applicable. NLEM, National List of Essential Medicines

Indicator	Value
Average number of drugs per encounter, mean ± SD	5.22 ± 1.37
Encounters with injections, N (%)	145 (24.2%)
Mean number of antibiotics per encounter, mean ± SD	0.44 ± 0.81
Drugs prescribed by generic name, N (%)	1,807 (57.7%)
Drugs prescribed from NLEM, N (%)	658 (21.0%)

Prevalence of polypharmacy and association with comorbidities

**Figure 1:**
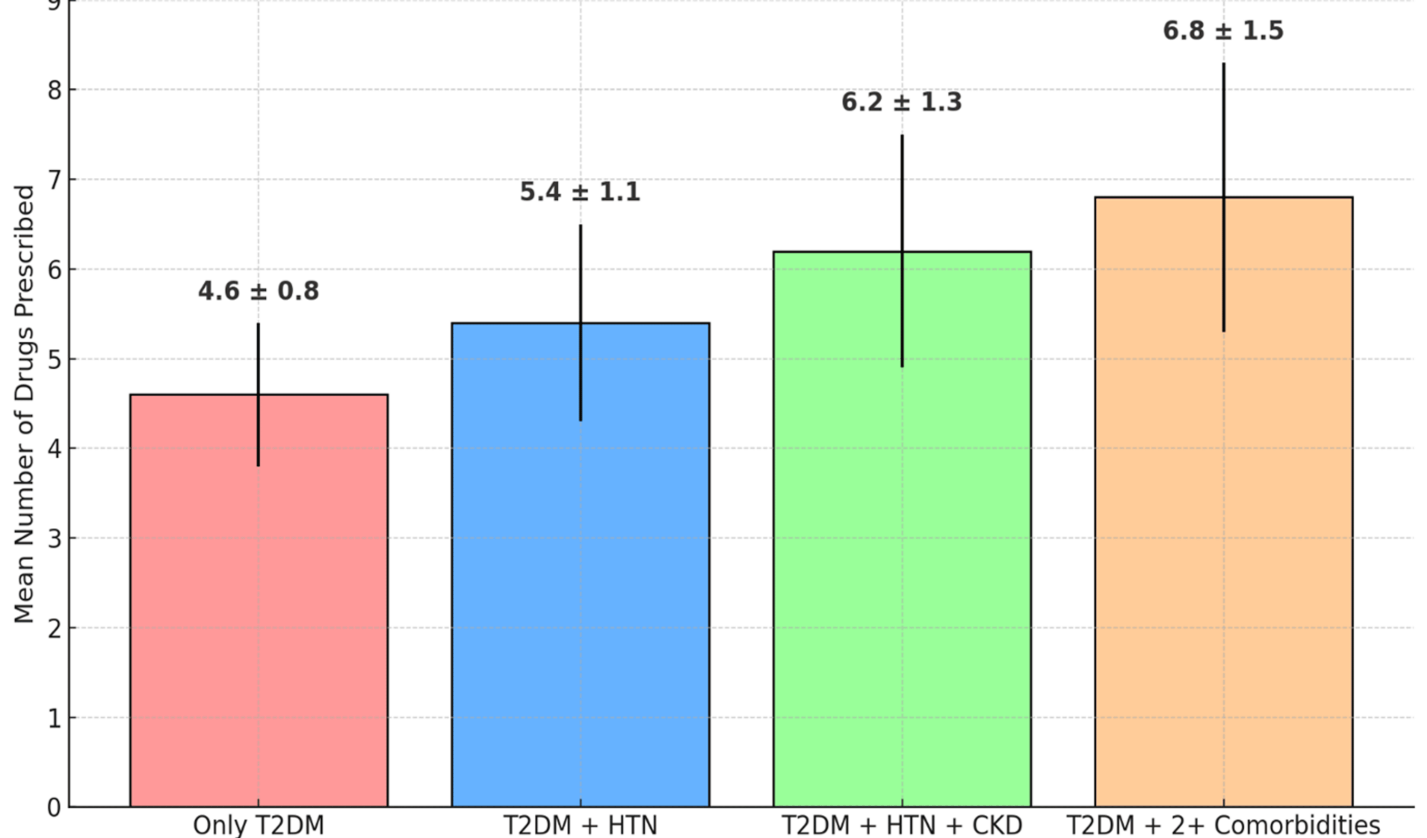
Polypharmacy across comorbidity groups in geriatric patients with type 2 diabetes mellitus (N = 600) Bars represent the mean ± standard deviation (SD) of the number of drugs prescribed per encounter.

The mean number of drugs per encounter varied by comorbidity status. Patients with only T2DM had a mean of 4.6 ± 0.8 drugs per encounter. Patients with one or more comorbidities had a higher mean number of drugs per encounter (Figure 1).

Pattern of antidiabetic drug utilization

**Figure 2:**
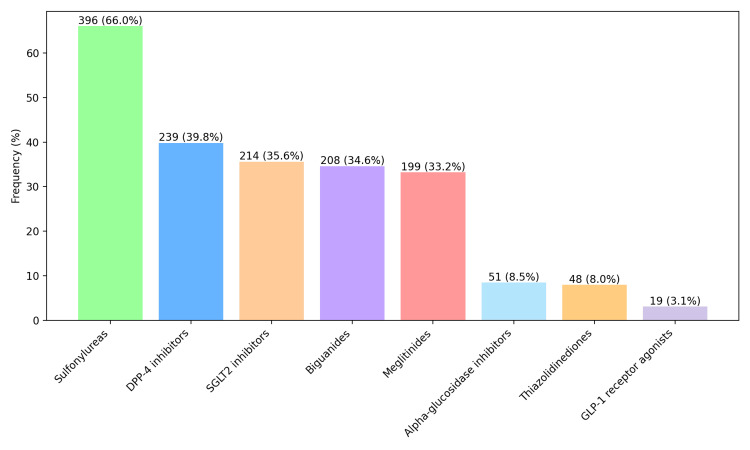
Distribution of antidiabetic drug classes prescribed (N = 600 encounters) Bars represent the number (percentage) (N (%)) of encounters in which each antidiabetic drug class was prescribed. Multiple drug classes could be prescribed per encounter.

Among antidiabetic drug classes, sulfonylureas were most frequently prescribed, followed by DPP-4 inhibitors. Alpha-glucosidase inhibitors, thiazolidinediones, and glucagon-like peptide-1 (GLP-1) receptor agonists were prescribed less frequently (Figure 2).

Pattern of antidiabetic therapy: monotherapy and combination therapy

**Figure 3:**
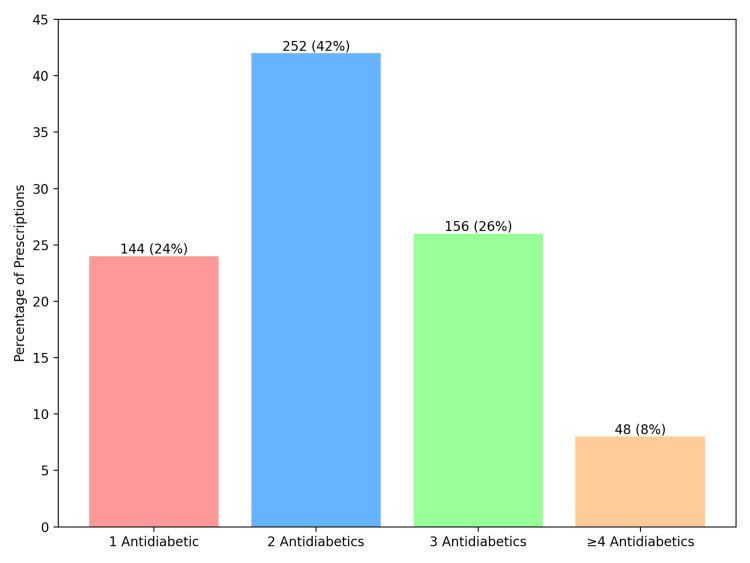
Number of antidiabetic drugs prescribed per encounter (N = 600 encounters). Bars represent the number (percentage) (N (%)) of encounters by therapy category.

Prescriptions containing one antidiabetic agent (monotherapy) accounted for 144 (24%) encounters, two antidiabetic agents (dual therapy) for 252 (42%) encounters, three antidiabetic agents (triple therapy) for 156 (26%) encounters, and four or more antidiabetic agents for 48 (8%) encounters (Figure 3).

## Discussion

The present study offers a detailed snapshot of real-world prescribing practices among geriatric patients with T2DM in a tertiary care teaching hospital. The findings underscore the clinical complexity of diabetes management in older adults, characterized by a high burden of comorbidities, substantial prevalence of polypharmacy, and variable adherence to principles of rational prescribing. These observations highlight both the necessity of individualized therapy in elderly patients and the need for system-level interventions to optimize pharmacotherapy.

With a mean age in the mid-sixties and a male preponderance, the study population's demographic profile is similar to trends found in a number of regional and Indian studies that have observed greater rates of diagnosis and healthcare use among older males [1,14]. The high prevalence of comorbidities such as hypertension, chronic kidney disease, and dyslipidemia reflects the clustering of cardiometabolic risk factors in older adults with diabetes [4]. Multimorbidity necessitates the concurrent use of multiple pharmacological agents, contributing to the high prevalence of polypharmacy observed in this study. While polypharmacy is often clinically unavoidable in this context, its risks must be carefully weighed against potential benefits.

The average number of drugs per prescription observed in the present study is consistent with values reported from tertiary care centers in India and other low- and middle-income countries [5,6]. However, polypharmacy has clinical ramifications that go beyond numbers. Adverse drug responses, drug-drug interactions, medication non-adherence, cognitive impairment, functional decline, and an increase in hospitalization among older populations have all been repeatedly linked to polypharmacy [9,15]. In patients with diabetes, these risks are further compounded by the potential for hypoglycemia, renal dysfunction, and cardiovascular events associated with certain antidiabetic and concomitant medications. Regular medication review, identification of potentially inappropriate medications, and deprescribing where clinically appropriate are therefore crucial components of geriatric diabetes care.

In the current study, slightly more than half of all prescription drugs were generics. Although this represents moderate adherence to rational prescribing norms, it remains below the optimal levels recommended by the WHO [16]. The variation in generic prescribing rates among studies could be due to a variety of factors, including patient preferences, institutional procurement regulations, pharmaceutical marketing effects, and prescriber assessments of drug quality. Improving affordability and adherence, especially for older patients on long-term multidrug regimens, may be achieved by fortifying institutional policies that support generic prescribing, making sure hospital pharmacies carry quality-assured generics, and educating prescribers about the financial advantages of generics.

The low proportion of medications prescribed from the NLEM observed in this study raises important concerns regarding adherence to national policy frameworks intended to promote cost-effective, evidence-based, and rational prescribing [17]. In public healthcare systems, the NLEM serves as a key tool for guiding prescribing and procurement practices. Several factors may contribute to the low utilization of essential medicines, including formulary restrictions, prescribers’ preference for newer branded medications, and limited awareness of essential medicine policies among clinicians. In addition, frequent use of newer antidiabetic agents, such as DPP-4 inhibitors and SGLT-2 inhibitors, which are commonly prescribed in tertiary care settings and may not be included in the specific version of the NLEM used for this audit, may partly explain the lower apparent adherence. Furthermore, tertiary care hospitals manage patients with complex comorbidities and complications, which may necessitate the use of non-NLEM medications based on clinical judgment and institutional formularies. Bridging this gap between policy and practice may be facilitated by integrating essential medicine concepts into continuing medical education, aligning institutional formularies with the NLEM, and conducting regular prescription audits.

The necessity for insulin therapy or other injectable drugs in older patients with inadequate glycemic control or contraindications to oral medications is reflected in the utilization of injectable therapies in over 25% of encounters. Even though injectable treatments are frequently therapeutically recommended, using them in elderly individuals poses particular difficulties because of issues with adherence, administration technique, hypoglycemia risk, and caregiver dependence [18]. Structured patient education, caregiver involvement, and regular follow-up are therefore essential to ensure safe and effective use of injectables in this population.

With respect to antidiabetic drug utilization, the predominance of sulfonylureas observed in this study likely reflects their low cost and widespread availability. However, the risk of hypoglycemia associated with sulfonylureas is well documented, particularly in elderly patients and those with renal impairment [19]. The relatively lower use of metformin (biguanides), despite its status as first-line therapy for T2DM, warrants contextual interpretation, as a substantial proportion of the study population had chronic kidney disease (35.1%), in whom metformin is contraindicated or requires dose adjustment depending on the stage of renal impairment. In routine tertiary care practice, clinicians may therefore preferentially prescribe alternative agents with a safer renal profile, such as DPP-4 inhibitors, and may rely on sulfonylureas due to their affordability and availability within institutional formularies. Contemporary guidelines increasingly emphasize the use of agents with a lower risk of hypoglycemia in older adults, such as DPP-4 inhibitors and, when appropriate, SGLT-2 inhibitors; however, the relatively limited use of newer agents may be influenced by cost considerations and access constraints in public healthcare settings. The increasing use of DPP-4 inhibitors observed in the present study may reflect a gradual shift toward safer therapeutic choices. Overall, these findings highlight how, in routine practice, clinical efficacy and safety must be balanced with affordability and accessibility, particularly in resource-constrained public healthcare settings.

The high utilization of sulfonylureas observed in the present study has important clinical implications in geriatric patients, as this drug class is associated with an increased risk of hypoglycemia, falls, and related morbidity in older adults, particularly in those with renal impairment and multiple comorbidities. Such adverse events can lead to emergency visits and hospitalizations, thereby increasing healthcare utilization and costs. In parallel, the low adherence to the NLEM raises concerns regarding both patient safety and economic burden, as deviation from essential medicine recommendations may increase out-of-pocket expenditure and reduce access to cost-effective therapies in public healthcare settings. Similar patterns of polypharmacy and preferential use of sulfonylureas in elderly patients with T2DM have been reported in other Indian and regional studies, whereas studies from high-income settings report greater use of newer antidiabetic agents with lower hypoglycemia risk, reflecting differences in resource availability and healthcare financing models. These comparisons highlight how prescribing patterns are shaped by both clinical complexity and health system constraints, underscoring the need for context-specific strategies to optimize medication safety, rational prescribing, and affordability in geriatric diabetes care.

The predominance of dual antidiabetic therapy in this study aligns with current recommendations advocating stepwise intensification of treatment to achieve glycemic targets when monotherapy is insufficient [20]. In contrast to a universal pursuit of stringent glycemic control, the intensification of therapy for senior patients should be guided by their specific glycemic targets, comorbidity burden, life expectancy, and patient preferences. Excessively aggressive glycemic objectives highlight the need for individualized treatment goals by raising the risk of hypoglycemia and unfavorable outcomes in fragile elderly patients.

Assessment of rationality was limited to WHO core prescribing indicators, and analytical linking of prescribing patterns to individual comorbidities was beyond the predefined descriptive scope of this study.

The results of this study demonstrate the need for regular prescription audits and drug utilization studies as instruments for quality enhancement from the standpoint of health systems. Including pharmacists and clinical pharmacologists in multidisciplinary care teams may improve medication safety in older populations, optimize drug regimens, and identify potentially inappropriate drugs. Reasonable drug use behaviors should be further reinforced by educational initiatives aimed at prescribers and institutional rules that support the prescription of generic and necessary medications. Evidence-based prescribing may be facilitated at the policy level by guaranteeing constant availability of indicated drugs and coordinating hospital formularies with national essential medicine lists.

The present findings provide insights into real-world prescribing practices in a tertiary care public hospital setting, highlighting how clinical decision-making in routine practice is influenced by patient complexity, comorbidity burden, and health system constraints. The observed high prevalence of polypharmacy reflects the need to manage multiple coexisting conditions in geriatric patients, while the pattern of antidiabetic drug utilization demonstrates how clinicians balance safety considerations (e.g., renal impairment and hypoglycemia risk) with drug availability and affordability. The relatively low adherence to the NLEM further underscores the gap that can exist between policy frameworks and everyday clinical practice in resource-constrained settings. Together, these findings illustrate the realities of real-world prescribing, where guideline recommendations are adapted to individual patient profiles and local healthcare system factors.

Strengths and limitations

This study’s strengths include its comparatively large sample size and methodical assessment of prescribing practices in a real-world tertiary care setting using standardized WHO indicators. The study identifies areas for focused intervention and provides useful insights into existing prescribing patterns in geriatric patients with T2DM. However, the cross-sectional design precludes evaluation of causal relationships and temporal changes in prescribing behavior. In addition, as the study was designed a priori as a descriptive drug utilization audit, inferential statistical analyses to explore associations between polypharmacy and clinical variables were not performed, which limits the ability to identify predictors of polypharmacy. The single-center design may restrict generalizability to other healthcare settings, particularly primary care or private healthcare facilities. Furthermore, the clinical significance of the observed prescribing patterns could not be fully assessed, as clinical outcomes such as glycemic control, incidence of hypoglycemia, adverse drug reactions, and patient adherence were not evaluated. Future multicenter and longitudinal studies incorporating analytical designs, clinical outcomes, and patient-reported measures are warranted to provide a more comprehensive understanding of the implications of prescribing practices in geriatric diabetes care.

## Conclusions

This study identifies significant trends in the prescribing of medications to geriatric patients with T2DM attending a tertiary care teaching hospital. Polypharmacy was highly prevalent, reflecting the substantial burden of comorbidities in this population. Although prudent use of antibiotics and modest levels of generic prescribing were observed, adherence to the NLEM was suboptimal. The continued reliance on older antidiabetic drug classes underscores the need for careful consideration of safety profiles, particularly with regard to hypoglycemia risk in elderly patients, while the predominance of combination antidiabetic therapy reflects the clinical complexity and challenges of achieving glycemic control in this age group.

These findings underscore the importance of implementing systematic medication review strategies in routine clinical practice, including periodic prescription audits, identification of potentially inappropriate medications, and consideration of deprescribing where clinically appropriate. Adoption of multidisciplinary approaches involving physicians, clinical pharmacologists, and pharmacists may further optimize pharmacotherapy and enhance medication safety. At the institutional level, aligning hospital formularies with the NLEM and strengthening continuing medical education on rational prescribing principles may improve adherence to essential medicine policies and promote cost-effective care. Collectively, these measures can directly inform improvements in geriatric diabetes management by enhancing medication safety, affordability, and quality of care. Future research should focus on evaluating the impact of multidisciplinary interventions, structured medication review strategies, and targeted prescribing policies on clinical outcomes, medication adherence, and quality of life in older adults with diabetes.

## References

[1] Pradeepa R, Mohan V (2021). Epidemiology of type 2 diabetes in India. Indian J Ophthalmol.

[2] Kirkman MS, Briscoe VJ, Clark N (2012). Diabetes in older adults. Diabetes Care.

[3] Sinclair AJ, Abdelhafiz A, Dunning T (2018). An international position statement on the management of frailty in diabetes mellitus: summary of recommendations 2017. J Frailty Aging.

[4] Indu R, Adhikari A, Maisnam I, Basak P, Sur TK, Das AK (2018). Polypharmacy and comorbidity status in the treatment of type 2 diabetic patients attending a tertiary care hospital: an observational and questionnaire-based study. Perspect Clin Res.

[5] Maher RL, Hanlon J, Hajjar ER (2014). Clinical consequences of polypharmacy in elderly. Expert Opin Drug Saf.

[6] Wastesson JW, Morin L, Tan EC, Johnell K (2018). An update on the clinical consequences of polypharmacy in older adults: a narrative review. Expert Opin Drug Saf.

[7] Mangoni AA, Jackson SH (2004). Age-related changes in pharmacokinetics and pharmacodynamics: basic principles and practical applications. Br J Clin Pharmacol.

[8] World Health Organization (1993). How to Investigate Drug Use in Health Facilities: Selected Drug Use Indicators. https://iris.who.int/items/87a82e02-a5a8-4539-b218-375061ff974c.

[9] Ofori-Asenso R (2016). A closer look at the World Health Organization's prescribing indicators. J Pharmacol Pharmacother.

[10] International Diabetes Federation (2026). International Diabetes Federation. The Diabetes Atlas. https://diabetesatlas.org.

[11] (2026). World Health Organization. WHO Model List of Essential Medicines - 23rd list, 2023. https://www.who.int/publications/i/item/WHO-MHP-HPS-EML-2023.02.

[12] Wiviott SD, Raz I, Bonaca MP (2019). Dapagliflozin and cardiovascular outcomes in type 2 diabetes. N Engl J Med.

[13] Singh A, Mohamedali SP, Ansari M (2018). Pattern of management of febrile neutropenia among breast cancer patients treated with different chemotherapeutic regimens. Int J Basic Clin Pharmacol.

[14] Hasan MS, Ghosal S (2023). Gender differentials in the choice of in-patient healthcare services among the older adults in India: a cross-sectional study. Int J Health Plann Manage.

[15] Kumari S, Jain S, Kumar S (2022). Effects of polypharmacy in elderly diabetic patients: a review. Cureus.

[16] Doni K, Bühn S, Weise A (2022). Safety of dipeptidyl peptidase-4 inhibitors in older adults with type 2 diabetes: a systematic review and meta-analysis of randomized controlled trials. Ther Adv Drug Saf.

[17] Roy V, Rana P (2018). Prescribing generics: all in a name. Indian J Med Res.

[18] (2026). World Health Organization. Medicines. https://www.who.int/health-topics/medicines.

[19] (2026). Government of India, Ministry of Health and Family Welfare. National List of Essential Medicines (NLEM). https://www.mohfw.gov.in/.

[20] Valencia WM, Florez HJ, Palacio AM (2019). Suitable use of injectable agents to overcome hypoglycemia risk, barriers, and clinical inertia in community-dwelling older adults with type 2 diabetes mellitus. Drugs Aging.

